# Unraveling HIV-1 spread in Southwest China: a phylogenetic and molecular network approach

**DOI:** 10.3389/fmicb.2026.1761072

**Published:** 2026-03-09

**Authors:** Xianwu Pang, Kailing Tang, Qin He, Jie Ma, Ningye Fang, Haomin Xie, Ge Zhong, Shujia Liang

**Affiliations:** Guangxi Key Laboratory for the Prevention and Control of Viral Hepatitis, Guangxi Key Laboratory of AIDS Prevention and Control and Achievement Transformation, Guangxi Zhuang Autonomous Region Center for Disease Control and Prevention, Nanning, Guangxi, China

**Keywords:** HIV-1, molecular epidemiology, molecular transmission networks, spatial epidemiology, transmission dynamics

## Abstract

Guangxi is one of the regions most affected by HIV-1 in China, yet the fine-scale transmission dynamics and molecular epidemiology remain incompletely characterized. In this study, we integrated molecular transmission network analysis, phylogenetic inference, and spatial analysis to elucidate HIV-1 dispersal patterns and inform precision public health interventions. We analyzed 10,199 HIV-1 pol sequences collected from all 14 cities in Guangxi, encompassing major subtypes including CRF01_AE, CRF07_BC, CRF08_BC, and CRF55_01B. Molecular networks were constructed using a 1.5% TN93 genetic-distance threshold, and logistic regression was applied to identify factors associated with network clustering. Bayesian phylogenetic and phylogeographic analyses were used to characterize spatiotemporal dissemination patterns. Overall, 75.6% (7,706/10,199) of individuals clustered within molecular networks, with clustering proportions exceeding 60% in every city. Factors independently associated with clustering included viral load >10,000 copies/mL (OR = 1.23, 95% CI: 1.10–1.39), education level of junior high school or below (OR = 1.67, 95% CI: 1.30–2.14), age ≥50 years (OR = 1.38, 95% CI: 1.16–1.63), Zhuang ethnicity (OR = 1.18, 95% CI: 1.08–1.30), and syringe sharing (OR = 1.65, 95% CI: 1.14–2.38). Intercity connections accounted for 48.2% of inferred genetic linkages, with CRF55_01B showing the highest intercity connectivity (60.4%). CRF01_AE displayed the broadest geographic distribution, Nanning and Qinzhou emerged as key connectivity hubs. Phylogeographic analyses suggested that Qinzhou was a major source of dispersal for CRF01_AE and CRF08_BC, whereas Nanning played a central role in the dissemination of CRF07_BC, CRF08_BC, and CRF55_01B. HIV-1 transmission in Guangxi is characterized by high network clustering and pronounced spatial heterogeneity, with distinct hub cities contributing to regional connectivity. These findings provide actionable evidence to support targeted, location-specific HIV prevention and control strategies at both local and regional levels.

## Introduction

Acquired Immunodeficiency Syndrome (AIDS) remains a major global public health challenge (Huang et al., 2015; The Lancet, 2017). Beyond individual-level risk, controlling HIV requires understanding where transmission concentrates and how infections disseminate across geographic areas, because intercity mobility can connect local epidemics, sustain transmission chains, and undermine city-level prevention efforts. Identifying spatial transmission hotspots, hub cities, and dissemination pathways is therefore essential for designing precision public health interventions and optimizing resource allocation.

Guangxi, a southwestern border province of China with approximately 47 million residents, bears one of the country’s highest HIV burdens. Since the epidemic was first identified in 1996, transmission patterns have shifted from being driven largely by injecting drug use to predominantly heterosexual transmission, alongside pronounced regional disparities and substantial rural impact (Chinese Center for Disease Control and Prevention, 2007; Chinese Center for Disease Control and Prevention, 2003; Chinese Center for Disease Control and Prevention, 2006; Chen et al., 2019; Ge et al., 2019). While traditional epidemiological approaches describe population-level trends, they are limited in resolving fine-scale spatial transmission pathways and intercity dissemination due to HIV’s long asymptomatic period and social reporting barriers (Kazanjian, 2017). Importantly, comprehensive province-wide evidence delineating HIV-1 spatial spread across all cities in Guangxi remains scarce.

Recent advances in molecular epidemiology have enabled more detailed reconstruction of HIV transmission dynamics. Genetic distance–based molecular transmission networks can reveal hidden clustering patterns and potential transmission linkages, complementing conventional field investigations (Smith et al., 2009; Aldous et al., 2012; Volz et al., 2018; Zhang et al., 2022; Zhang J. et al., 2023; Paraskevis et al., 2019; Zhang et al., 2017). When integrated with phylogenetic and Bayesian phylogeographic approaches, these methods can further infer spatial dissemination directionality and quantify migration among locations (Stecher et al., 2019; Chaillon et al., 2017; Zhang F. et al., 2023). The continued evolution of molecular network methodologies has provided novel insights for HIV/AIDS prevention and control (Wertheim et al., 2019; Chang et al., 2018), a point underscored in 2018 when the U.S. Centers for Disease Control and Prevention (CDC) formally recognized the utility of these approaches for identifying undiagnosed infections and high-risk populations (Oster et al., 2018). When combined with conventional epidemiological investigations, molecular network analysis offers a precise tool for optimizing public health interventions (Ragonnet-Cronin et al., 2019).

Such integrated genomic–spatial frameworks have improved identification of transmission hubs in multiple settings, yet they have not been systematically applied to map subtype-specific spatial dissemination across all 14 cities in Guangxi. Here, we combine molecular transmission network analysis, phylogenetic inference, and spatial modeling to comprehensively characterize HIV-1 transmission and intercity spread in Guangxi, identify key hubs and high-risk groups, and inform precision-targeted interventions.

## Materials and methods

### Study design and participants

A cross-sectional study was conducted in Guangxi, a southwestern province of China bordering Vietnam and heavily affected by the HIV/AIDS epidemic. We analyzed HIV-1 pol sequences obtained through the national HIV drug-resistance surveillance program, which routinely collects samples from people living with HIV (PLWH) experiencing virologic failure after antiretroviral therapy (ART). Samples collected between January 2019 and June 2024 from all 14 cities in Guangxi were included. Before analysis, duplicate and incomplete records were excluded to ensure data uniqueness and comparability. Importantly, all eligible samples meeting national surveillance and quality-control criteria during the study period were included. The inclusion and exclusion process of study participants is summarized in Supplementary Figure S1.

This study received ethical approval from the Ethics Review Committee of the Guangxi Center for Disease Control and Prevention. Written informed consent was obtained from all participants, allowing the use of their samples, demographic information, and clinical records for epidemiological research, including age, gender, ethnicity, marital status, syringe sharing, extramarital sexual activity, viral load, CD4+ T lymphocyte count, and infection route.

### Sequences processing and HIV-1 subtyping

Plasma samples were collected from multiple cities and maintained under cold-chain conditions before transport to the Guangxi CDC laboratory. HIV RNA extraction, nested PCR amplification of the pol region (HXB2: 2147–3,462), sequencing, and sequence quality control were performed following protocols previously described by our group (Pang et al., 2024). Briefly, viral RNA was extracted using the QIAamp Viral RNA Kit (Qiagen, Hilden, Germany), and the pol region was amplified by nested PCR. Sequences were edited using Sequencher v5.1 and aligned with BioEdit v7.1. HIV-1 subtypes were determined by phylogenetic analysis through comparison with reference sequences obtained from the Los Alamos HIV Sequence Database, as described previously.

### HIV transmission network construction

Pairwise genetic distances among aligned sequences were calculated using the Tamura-Nei 93 (TN93) model in HyPhy software, in accordance with the National Technical Guideline for HIV Transmission Network Monitoring and Intervention in China. Sensitivity analyses were conducted across a range of genetic-distance thresholds (0.3%–2.1%) to assess network stability and identify the optimal cutoff based on the number of detected clusters, nodes, and link intersections (Supplementary Figure S2). Finally, the threshold of 1.5% was selected as optimal for defining potential transmission clusters based on previously published large-scale molecular epidemiological studies conducted in China and national technical guidelines (Yuan et al., 2021; Zhang F. et al., 2023). Network visualization was performed using Cytoscape v3.5.1.

### Bayesian phylogenetic and Phylogeographic analysis

Spatial distribution and clustering of five HIV-1 subtypes across Guangxi were assessed by calculating the number of PLWH per subtype and their respective clustering rates, defined as the proportion of individuals within molecular transmission networks. Intercity connectivity was quantified as the proportion of inferred genetic linkages occurring between cities relative to all network connections and visualized using numerical matrices with color-coded intensity. Intracity heterogeneity in transmission routes was evaluated using the Pielou index, calculated as: $-\sum_{i=I}^{s}\mathrm{pi}\times \ln\;\mathrm{pi}/\ln\;S$ , where pi represents the proportion of each transmission route and S denotes the total number of transmission routes (five in this study). The index ranges from 0 (complete homogeneity) to 1 (complete heterogeneity).

Bayesian phylogenetic analysis was conducted to infer HIV-1 spatial dispersal and migration events among cities in Guangxi. Maximum-likelihood phylogenetic trees were first reconstructed using FastTree based on the HIV-1 pol sequences. Temporal signal was subsequently assessed using TempEst v1.5.3, and only datasets exhibiting a positive root-to-tip correlation (correlation coefficient ≥0.3) were considered suitable for molecular clock–based inference. Bayesian analyses were performed in BEAST v1.10.5 using the generalized time-reversible (GTR) substitution model with an uncorrelated log-normal relaxed molecular clock under a Skygrid coalescent prior. Markov chain Monte Carlo (MCMC) analyses were conducted separately for major subtypes, with chain lengths of 50 million states for CRF01_AE, 100 million for CRF07_BC, 200 million for CRF08_BC, and 400 million for CRF55_01B. Parameters were sampled every 10,000 steps, and the first 10% of samples were discarded as burn-in. Convergence and adequate mixing were assessed using Tracer v1.7.2, with effective sample size (ESS) values ≥200 indicating sufficient convergence. Maximum clade credibility (MCC) trees were generated after removal of burn-in. Spatial dispersal patterns were further characterized using a Bayesian stochastic search variable selection (BSSVS) approach, and the expected number of viral migration events between cities was quantified using a robust counting (Markov jump) framework. Statistical support for inferred migration routes was evaluated using Bayes factors (BF) and posterior probabilities calculated in SpreaD3 v0.9.6, and only migration events with BF ≥ 3 and posterior probability ≥0.9 were retained for interpretation and visualization.

### Statistical analysis

Demographic and clinical characteristics were summarized as frequencies and percentages, with missing data classified as unknown. Associations between variables and network clustering were assessed using univariable and multivariable logistic regression analyses. Variables included demographic characteristics (age, sex, ethnicity, education, marital status), behavioral factors (transmission route, syringe sharing, sexual behaviors), and clinical indicators (viral load). Univariable logistic regression was first performed, and variables that were statistically significant (*p* < 0.05) or considered potential confounders were entered into the multivariable model. Results were reported as crude odds ratios (cOR), adjusted odds ratios (aOR), and 95% confidence intervals (CI). A two-sided *p* value <0.05 was considered statistically significant. All statistical analyses were conducted using R v4.4.3, and data visualizations were generated using GraphPad Prism v9 and Python v3.13.

## Results

### HIV-1 genetic transmission networks

**Figure 1:**
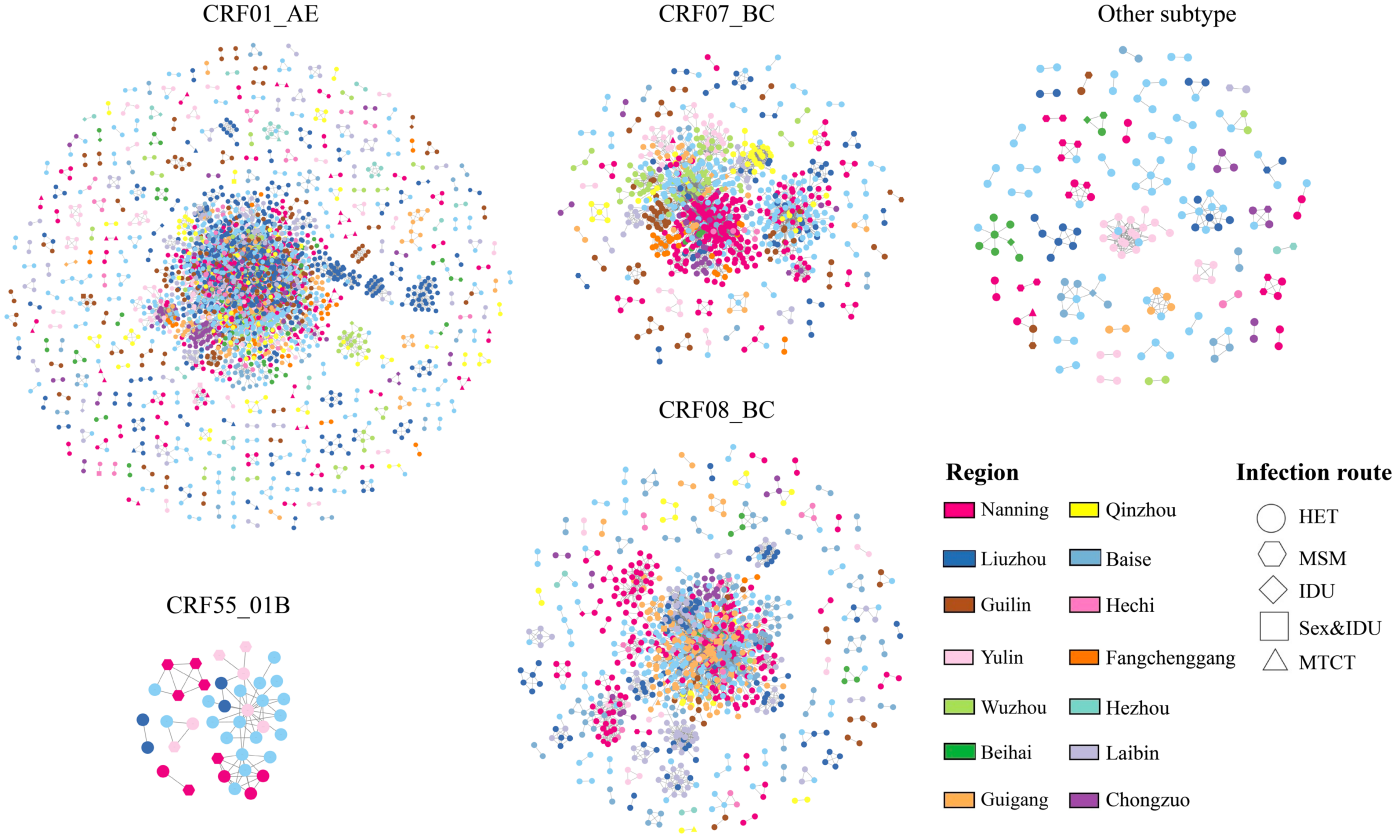
Molecular transmission network of HIV-1 subtypes. Each node represents an HIV-infected individual, and the lines indicate potential connections between them. Different colors represent 14 cities. Shapes denote different transmission routes: HET, heterosexual transmission; MSM, men who have sex with men; IDU, intravenous drug use; Sex&IDU, sexual contact and intravenous drug use; HTCT, mother-to-child transmission. The number of molecular clusters for CRF01_AE, CRF07_BC, CRF08_BC, CRF55_01B, and other subtypes are 301 (59.1%), 61 (12.0%), 89 (17.5%), 5 (1.0%), and 53 (10.4%), respectively.

A total of 10,199 people living with HIV (PLWH) were included in the analysis. The predominant HIV-1 subtypes were CRF01_AE (60.1%), CRF08_BC (19.9%), CRF07_BC (14.2%), CRF55_01B (0.9%), and other subtypes (4.9%). Subtype distributions showed marked geographic heterogeneity across the 14 cities. CRF08_BC predominated in Qinzhou and Baise, whereas CRF01_AE was dominant in most other cities (Supplementary Figure S3). Using a genetic-distance threshold of 1.5%, 7,706 individuals (75.6%) were connected to at least one other sequence, forming 509 molecular clusters (Figure 1). Subtype-specific clustering proportions were highest for CRF01_AE (78.5%), CRF08_BC (78.1%), and CRF07_BC (73.9%), whereas CRF55_01B (55.3%) and other subtypes (37.4%) exhibited substantially lower clustering (Supplementary Table S1).

### Determinants of HIV-1 molecular transmission network

Overall, males accounted for 71.7% (7,312/10,199) of the cohort, of whom 5,326 were included in molecular clusters. In univariable analysis, females showed a higher likelihood of clustering (OR = 1.18, *p* = 0.001); however, this association was no longer significant after multivariable adjustment (aOR = 1.03, *p* = 0.602). Ethnicity was independently associated with clustering. Compared with Han individuals (54.8%), Zhuang ethnicity (40.2%) was associated with a higher likelihood of clustering (aOR = 1.18, *p* = 0.001). Age was also a significant determinant: participants aged ≥50 years were more likely to cluster (aOR = 1.38, *p* < 0.001), whereas those aged 30–49 years showed no significant association relative to individuals <30 years (aOR = 0.94, *p* = 0.397). Educational attainment showed a strong gradient effect. Individuals with primary education or below had the highest likelihood of clustering (aOR = 1.81, *p* < 0.001), followed by those with junior high school education (aOR = 1.67, *p* < 0.001). After adjustment, neither injection drug use (IDU) nor men who have sex with men (MSM) was independently associated with clustering. Higher viral load was positively associated with clustering, with adjusted odds ratios of 1.23 (*p* = 0.001) for 10,000–50,000 copies/mL and 1.21 (*p* = 0.001) for >50,000 copies/mL, compared with <10,000 copies/mL. Syringe sharing with 1–4 partners was also independently associated with clustering (aOR = 1.65, *p* = 0.007), whereas non-marital heterosexual partnerships and same-sex partnerships were not significantly associated after adjustment (Table 1).

**Table 1:** Factors associated with clustering among PLWH.

Variables	Total, *n*(%)	In networks, *n*(%)	Undjusted OR	*p*-value	Adjusted OR	*p*-value
Gender						
Male	7,312 (71.7)	5,473 (71.0)	Ref		Ref	
Female	2,887 (28.3)	2,233 (29.0)	1.18 (1.07–1.30)	0.001	1.03 (0.92–1.16)	0.602
Ethnicity						
Han	5,587 (54.8)	4,135 (53.7)	Ref		Ref	
Zhuang	4,095 (40.2)	3,177 (41.2)	1.22 (1.11–1.34)	<0.001	1.18 (1.08–1.30)	0.001
Minority	517 (5.1)	394 (5.1)	1.11 (0.90–1.36)	0.339	1.14 (0.92–1.41)	0.221
Age						
< 30	1,683 (16.5)	1,192 (15.5)	Ref		Ref	
30–49	3,558 (34.9)	2,557 (33.2)	1.11 (0.98–1.26)	0.108	0.94 (0.81–1.09)	0.397
≥ 50	4,958 (48.6)	3,957 (51.3)	1.75 (1.55–1.98)	<0.001	1.38 (1.16–1.63)	<0.001
Marital status						
Unmarried	2,892 (28.4)	2067 (26.8)	Ref		Ref	
Married	5,074 (49.7)	3,905 (50.7)	1.38 (1.25–1.53)	<0.001	1.06 (0.94–1.21)	0.346
Divorced/widowed	2,233 (21.9)	1734 (22.5)	1.48 (1.31–1.68)	<0.001	1.09 (0.94–1.26)	0.278
Educational level						
College and above	395 (3.9)	234 (3.0)	Ref		Ref	
High school or technical school	793 (7.8)	538 (7.0)	1.56 (1.22–1.99)	<0.001	1.17 (0.90–1.52)	0.249
Junior high school	3,684 (36.1)	2,748 (35.7)	2.46 (1.99–3.03)	<0.001	1.67 (1.30–2.14)	<0.001
Primary and below	5,327 (52.2)	4,186 (54.3)	3.11 (2.52–3.82)	<0.001	1.81 (1.40–2.34)	<0.001
Infection route						
HET	8,767 (86.0)	6,697 (86.9)	Ref		Ref	
MSM	449 (4.4)	258 (3.3)	0.32 (0.26–0.38)	<0.001	0.33 (0.10–1.14)	0.079
IDU	642 (6.3)	476 (6.2)	0.94 (0.79–1.13)	0.534	0.82 (0.59–1.15)	0.244
Sex&IDU	103 (1.0)	74 (1.0)	0.86 (0.56–1.33)	0.496	0.76 (0.45–1.29)	0.313
MTCT	238 (2.3)	201 (2.6)	1.78 (1.25–2.52)	0.001	1.54 (0.88–2.68)	0.128
Occupation						
Farmer	7,014 (68.8)	5,407 (70.2)	Ref		Ref	
Employed	2,511 (24.6)	1786 (23.2)	0.72 (0.65–0.79)	<0.001	0.98 (0.87–1.10)	0.734
Retired	201 (2.0)	156 (2.0)	1.09 (0.78–1.52)	0.607	1.11 (0.78–1.56)	0.566
Student/Child	361 (3.5)	276 (3.6)	0.83 (0.66–1.06)	0.131	0.99 (0.66–1.49)	0.959
Unknown	112 (1.1)	81 (1.1)	0.78 (0.52–1.17)	0.224	1.01 (0.66–1.55)	0.973
Viral load (copies/ml)						
<10,000	2,976 (29.2)	2,160 (28.0)	Ref		Ref	
10,000-50,000	2,703 (26.5)	2082 (27.0)	1.21 (1.07–1.36)	0.002	1.23 (1.10–1.39)	0.001
>50,000	4,520 (44.3)	3,464 (45.0)	1.20 (1.08–1.33)	0.001	1.21 (1.08–1.34)	0.001
Number of syringe sharers						
0	9,658 (94.7)	7,295 (94.7)	Ref		Ref	
1–4	425 (4.2)	331 (4.3)	1.21 (0.96–1.53)	0.105	1.65 (1.14–2.38)	0.007
≥ 5	116 (1.1)	80 (1.0)	0.80 (0.54–1.18)	0.257	1.06 (0.65–1.74)	0.822
Number of non-marital sex partners						
0	2,626 (25.7)	1956 (25.4)	Ref		Ref	
1–4	5,708 (56.0)	4,334 (56.2)	1.12 (1.01–1.24)	0.037	0.92 (0.80–1.06)	0.266
≥ 5	1865 (18.3)	1,416 (18.4)	1.17 (1.02–1.33)	0.026	0.96 (0.81–1.14)	0.621
Number of homosexual partners						
0	9,751 (95.6)	7,446 (96.6)	Ref		Ref	
1–4	264 (2.6)	143 (1.9)	0.26 (0.20–0.33)	<0.001	0.87 (0.27–2.75)	0.809
≥ 5	184 (1.8)	117 (1.5)	0.44 (0.33–0.59)	<0.001	1.55 (0.48–5.03)	0.47

Logistic regression analysis was conducted to identify factors influencing the clustering of people living with HIV.

### Spatial characteristics of the HIV-1 molecular transmission network

**Figure 2:**
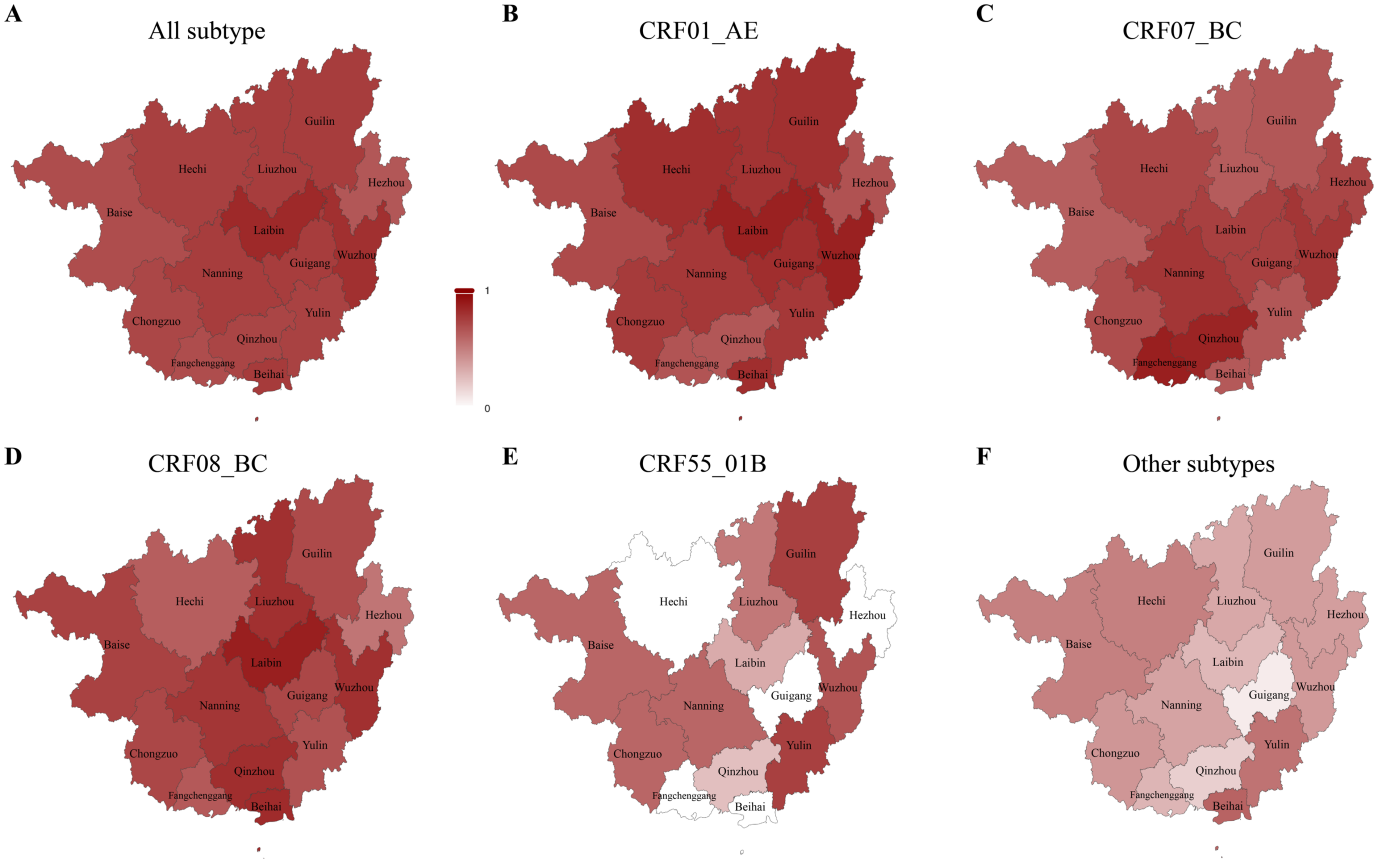
Spatial distribution of clustering rates for different HIV-1 subtypes. Smaller (larger) values indicate lower (higher) clustering rates, while larger values indicate higher clustering rates. **(A–F)** represent the spatial distribution of clustering rates for all subtypes, CRF01_AE, CRF07_BC, CRF08_BC, CRF55_01B, and other subtypes, respectively.

**Figure 3:**
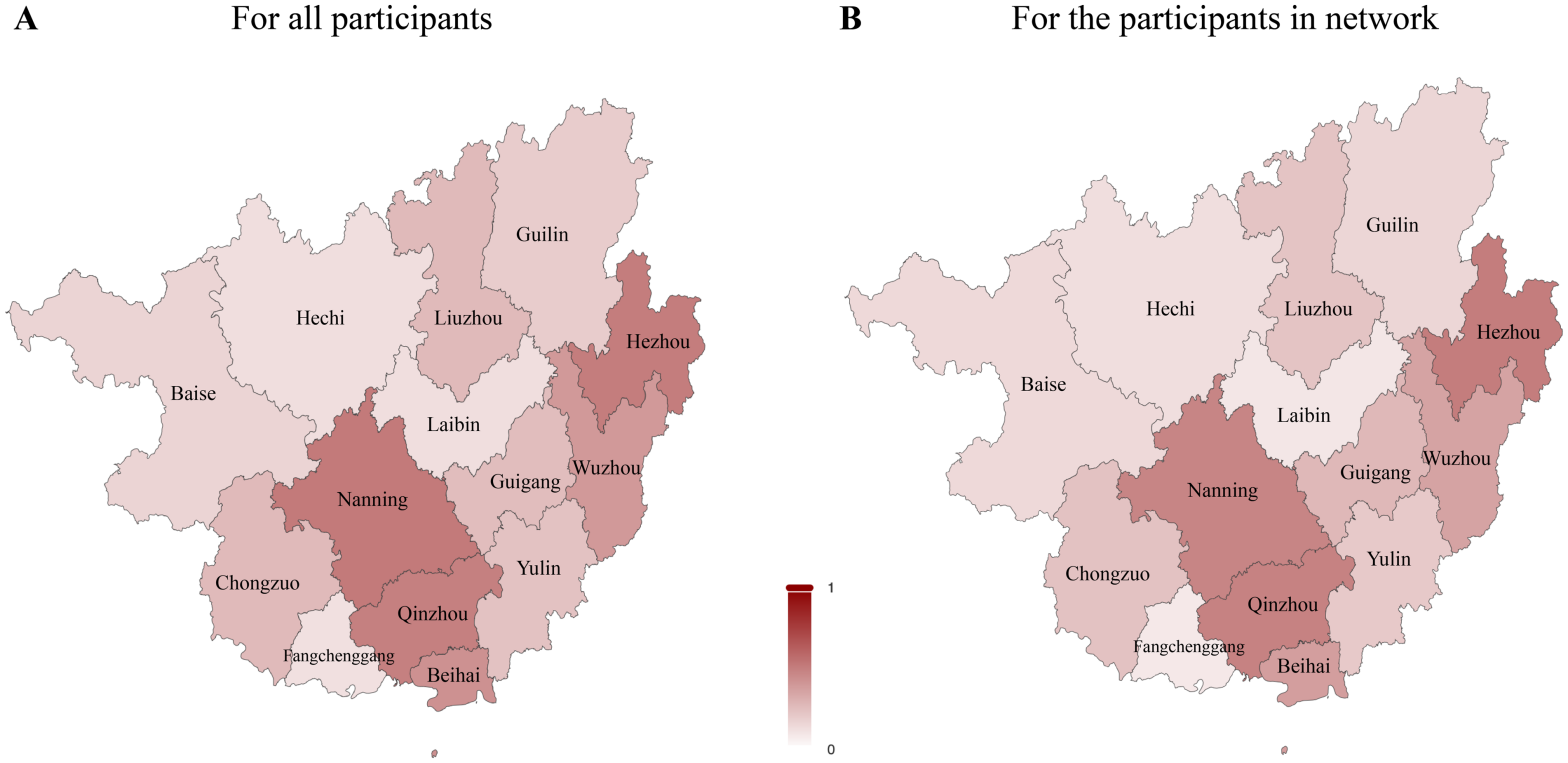
Heterogeneity of HIV transmission routes in each city represented by the Pielou index. A smaller Pielou index value indicates more homogeneity in HIV transmission routes within the city, while a larger Pielou index value indicates greater heterogeneity in HIV transmission routes. **(A,B)** represent Heterogeneity of HIV transmission routes for all participants, the participants in network, respectively.

Clustering proportions exceeded 60% in all 14 cities, indicating widespread network connectivity across the province. The highest clustering rates were observed in Laibin (84.3%) and Wuzhou (81.5%) (Supplementary Table S1). Subtype-specific clustering patterns varied geographically. For CRF01_AE, the highest clustering was observed in Wuzhou (87.1%), Laibin (86.9%), and Hechi (81.9%). CRF07_BC clustering was highest in Fangchenggang (88.1%), Qinzhou (86.1%), and Nanning (79.4%). CRF08_BC clustering peaked in Laibin (87.9%), Beihai (82.8%), and Qinzhou (81.6%). For CRF55_01B, the highest clustering was observed in Guilin and Yulin (75.0% each), while other subtypes clustered most frequently in Beihai (61.1%) and Yulin (55.1%) (Figure 2). Demographic composition and dominant transmission routes varied substantially across cities. Overall intercity linkage proportions were consistent with the general spatial distribution of molecular connections (Supplementary Figure S4). Cities exhibiting the greatest heterogeneity in transmission routes, as measured by the Pielou index, included Nanning (>0.52), Hezhou (>0.51), and Qinzhou (>0.50) (Figure 3).

### Genetic linkages within and between cities

**Figure 4:**
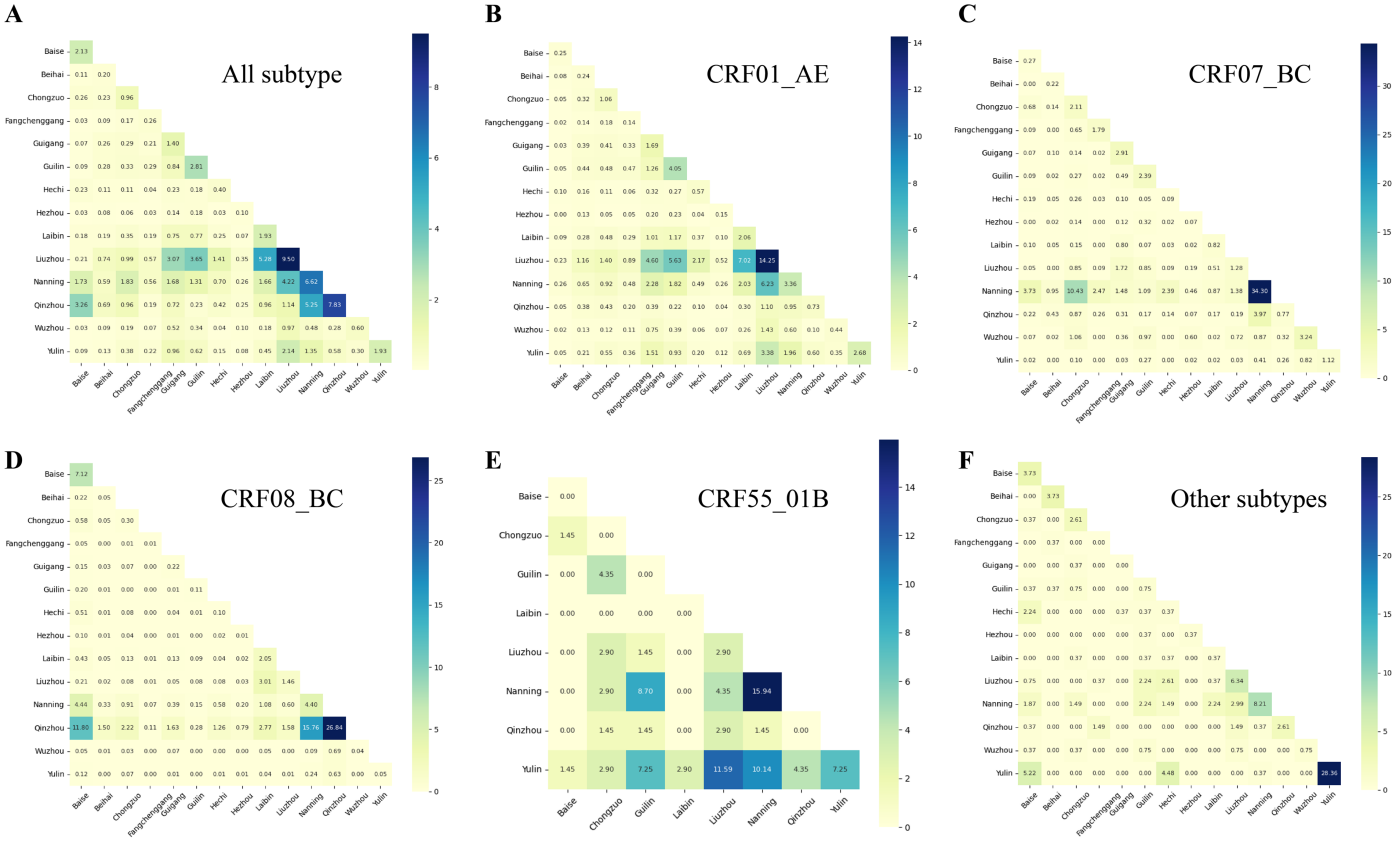
Strength matrix of HIV-1 transmission connections between cities in Guangxi. The value and color of the grid cell at the intersection of two cities indicate the number of connections between HIV-infected individuals (PLWH) in those cities. **(A–F)** Represent the strength matrices for all subtypes, CRF01_AE, CRF07_BC, CRF08_BC, CRF55_01B, and other subtypes, respectively.

Analysis of molecular network connectivity revealed strong intracity genetic linkages in most cities, alongside substantial intercity connectivity. The intensity matrix highlighted prominent intercity linkages between Nanning and Liuzhou, Nanning and Qinzhou, and Liuzhou and Guilin (Figure 4). Although most subtypes showed predominantly intracity connections, several exhibited notable intercity patterns. For CRF01_AE, strong intercity linkages were observed between Liuzhou and Nanning, as well as connections involving Guilin, Guigang, and Laibin. CRF07_BC intercity connectivity was largely concentrated between Nanning and Chongzuo. For CRF08_BC, the strongest intercity linkages were observed between Qinzhou and Nanning, and between Qinzhou and Baise. CRF55_01B exhibited notable intercity connectivity between Nanning and Guilin, as well as between Yulin and Liuzhou, Guilin, and Qinzhou. The proportion of intercity genetic linkages ranged from 0.40 in Beihai to 0.55 in Hezhou, with seven cities exhibiting intercity proportions exceeding 0.50 (Supplementary Table S5).

**Figure 5:**
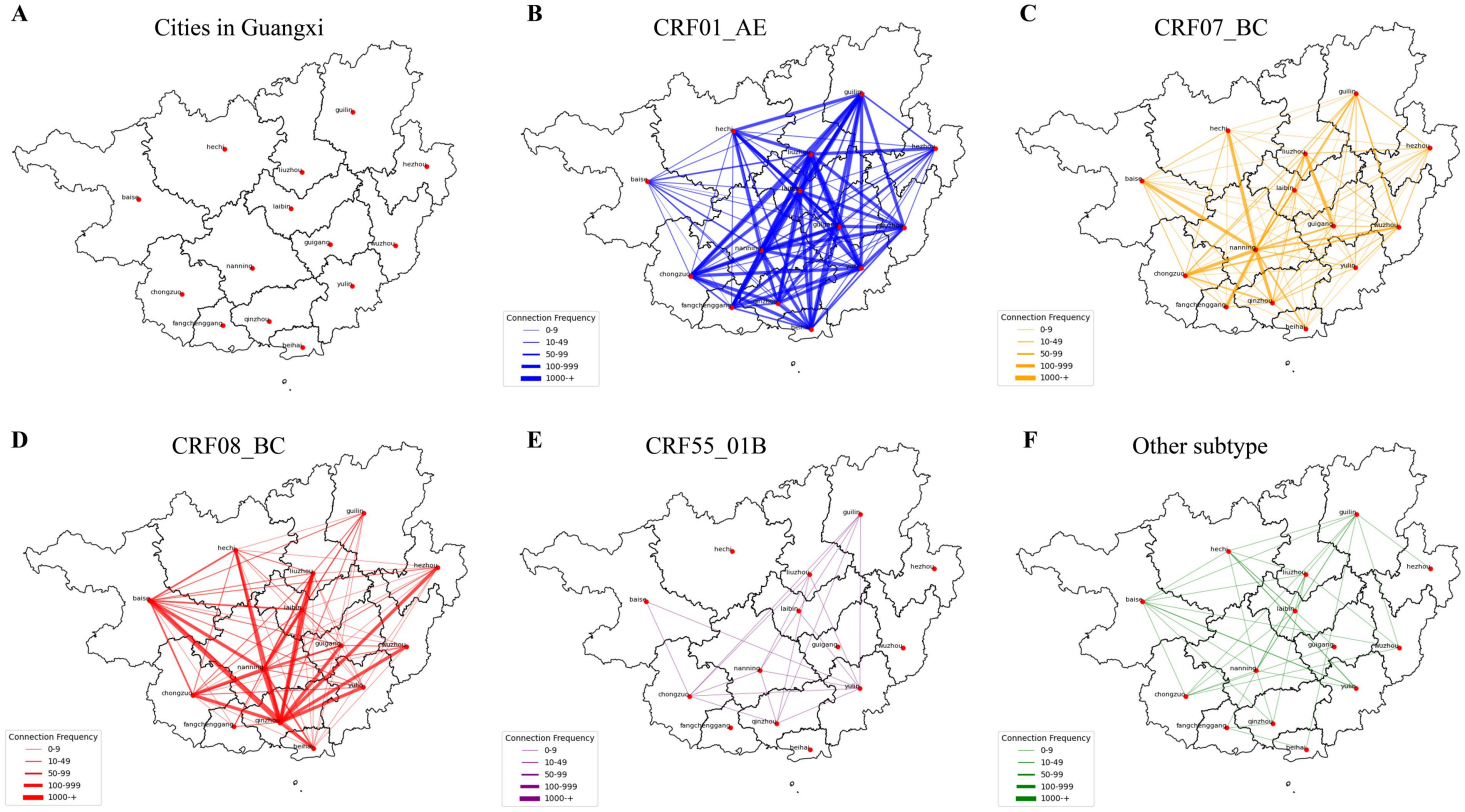
HIV transmission flow diagram in the molecular transmission network of HIV-1 between cities in Guangxi. Each line represents the HIV transmission connection between two cities, with each color indicating a different HIV-1 subtype. The thickness of the lines represents the number of connections between PLWH in the two cities. **(A)** Cities in Guangxi; **(B–F)** represent the transmission network for CRF01_AE, CRF07_BC, CRF08_BC, CRF55_01B, and other subtypes, respectively.

The spatial patterns of intercity genetic connectivity varied substantially among HIV-1 subtypes. CRF01_AE exhibited the most extensive intercity linkages, with particularly strong connections between Liuzhou and Nanning, followed by additional links involving Laibin, Guigang, and Guilin. CRF08_BC showed the second-highest level of intercity connectivity, with Nanning and Qinzhou acting as major connectivity hubs, and secondary linkages involving Laibin and Baise. In contrast, intercity linkages for CRF07_BC were largely concentrated between Nanning and Chongzuo, highlighting the central role of these cities within the CRF07_BC network. For CRF55_01B, the strongest intercity linkages were observed between Yulin and Nanning, with additional connections involving Guilin and Liuzhou, suggesting a more regionally confined connectivity pattern (Figure 5).

### Spatial and temporal spread of different subtypes among cities

**Figure 6:**
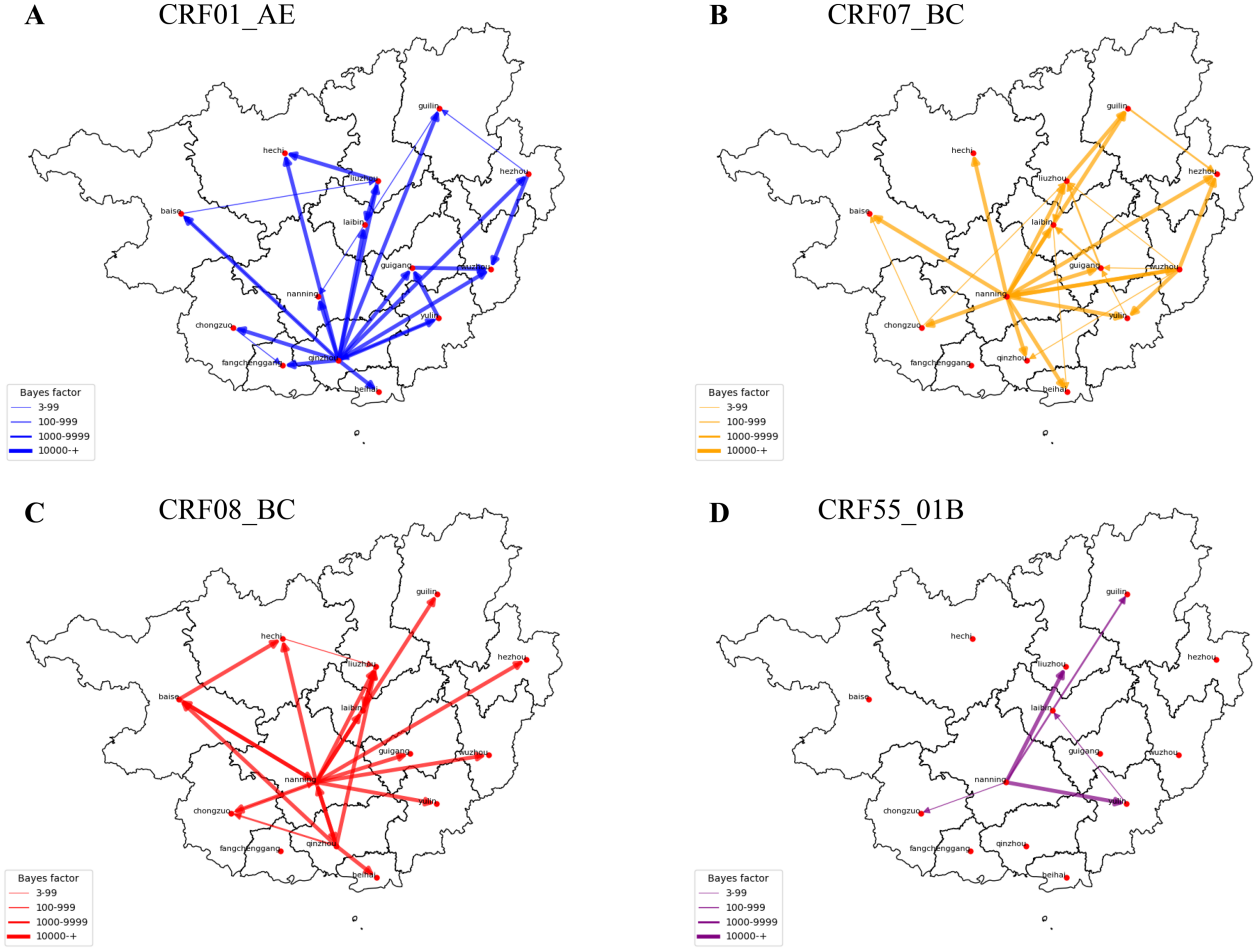
HIV migration events. Well-supported virus dispersal events among cities in Guangxi: **(A)** CRF01_AE, **(B)** CRF07_BC, **(C)** CRF08_BC, **(D)** CRF55_01B. Only results with a Bayes factor (BF) ≥ 3 and posterior probability support ≥0.9 are presented. Arrows indicate the direction of HIV migration events.

Bayesian phylogenetic analysis further elucidated the spatial and temporal dissemination patterns of major HIV-1 subtypes across Guangxi. For CRF01_AE, well-supported viral dispersal events were inferred from Qinzhou to 13 cities, followed by additional dissemination from Liuzhou to Hechi and Laibin, from Yulin to Guigang and Qinzhou, and from Hezhou to Wuzhou and Guilin. For CRF07_BC, Nanning emerged as the dominant dispersal hub, with inferred migration events to 13 cities, while Wuzhou also contributed to onward spread. For CRF08_BC, Nanning was the primary inferred source, with dispersal to 11 cities, followed by Qinzhou (six cities) and Baise (three cities). For CRF55_01B, the most frequent migration events were inferred from Nanning to Liuzhou, Guilin, and Yulin, with additional dispersal from Yulin to Laibin (Figure 6; Supplementary Table S5).

## Discussion

This study provides a province-wide, integrative analysis of HIV-1 genetic connectivity in Guangxi by combining molecular transmission network analysis, phylogenetic inference, and spatial epidemiology. By examining intercity linkage patterns across all 14 cities, we identified pronounced spatial heterogeneity, key connectivity hubs, and subtype-specific dissemination patterns, offering important insights for location-specific HIV prevention strategies.

Spatial network analysis revealed substantial intercity genetic connectivity, with nearly half (48.2%) of all inferred genetic links occurring between cities. Among the identified hubs, Qinzhou and Nanning played distinct yet complementary roles. Qinzhou emerged as a major source of inferred dispersal for CRF01_AE and CRF08_BC, whereas Nanning functioned as a central connectivity hub for multiple subtypes, including CRF07_BC, CRF08_BC, and CRF55_01B. These findings indicate that HIV-1 spread in Guangxi is not restricted to administrative boundaries but is shaped by regional connectivity and population mobility.

The prominent role of Qinzhou as an inferred upstream source is epidemiologically meaningful. Compared with other cities, Qinzhou has a higher proportion of injection drug use and a relatively elevated prevalence of mixed sexual and injection-related transmission, suggesting sustained overlap between traditional high-risk groups and the broader heterosexual population (Tao et al., 2013). Such overlap may facilitate onward viral dissemination to other cities, particularly for CRF01_AE and CRF08_BC. In contrast, Nanning, as the provincial capital, exhibited greater heterogeneity in transmission routes and extensive intercity connectivity, consistent with its role as a major transportation, economic, and migration center. Together, these findings suggest a spatial structure in which Qinzhou may act as a key upstream source, while Nanning functions as a downstream amplifier, underscoring the need for differentiated and city-specific intervention strategies.

Spatial variations in demographic composition further help explain the observed connectivity patterns. Several cities with high clustering proportions, such as Guilin, Laibin, and Wuzhou, have a relatively large share of individuals aged ≥50 years, a group independently associated with clustering in multivariable analysis. The observed association between older age (≥50 years) and molecular clustering is likely driven by behavioral and social factors rather than biological susceptibility alone. In Guangxi, older adults—particularly in rural settings—have been reported to engage in unprotected low-cost commercial sex and to use aphrodisiacs, often with limited awareness of HIV prevention and low perceived personal risk. Condom use in this age group is frequently inconsistent, and HIV testing may be delayed due to stigma or the misconception. These factors may facilitate sustained transmission within relatively stable and interconnected sexual networks, resulting in higher clustering proportions observed among older individuals. Similar age-related clustering patterns have been reported in previous molecular epidemiological studies conducted in Guangxi and other regions of China, supporting the plausibility of these mechanisms (Zhang F. et al., 2023). In addition, certain cities, including Qinzhou and Wuzhou, exhibited particularly high proportions of male cases, alongside marked heterogeneity in dominant transmission routes, with varying contributions from heterosexual contact and injection drug use. These demographic and behavioral differences are consistent with the spatial clustering and intercity connectivity observed in the molecular networks, supporting the interpretation that local population structure and prevailing transmission routes shape city-specific HIV epidemic dynamics.

At the individual level, the high overall clustering proportion (75.6%) suggests that HIV-1 spread in Guangxi predominantly occurs within interconnected networks rather than as isolated events. Older adults (≥50 years), individuals with lower educational attainment, and those with higher viral loads were more likely to be part of molecular clusters (Zhang F. et al., 2023). In Guangxi, older individuals, particularly in rural areas, may engage in unprotected low-cost commercial sex or have limited access to HIV prevention information, increasing vulnerability to infection (Tang et al., 2014). The elevated clustering observed among individuals of Zhuang ethnicity further indicates that cultural, socioeconomic, and geographic factors intersect to influence HIV transmission dynamics. These findings highlight the importance of age-specific and culturally tailored prevention strategies, particularly in rural and minority communities.

From a public health perspective, our results provide actionable guidance for precision HIV intervention. Previous studies have shown that increased contact rates, population mobility, and diverse transmission routes can amplify viral spread in urban settings (Alirol et al., 2011). Cities often function as epidemic amplifiers, and ongoing urbanization may further strengthen intercity connectivity. Accordingly, international organizations such as the Global Fund, WHO, and UNAIDS advocate for geographically targeted HIV prevention approaches (Ratmann et al., 2020). Although molecular epidemiological inferences are constrained by incomplete sampling and the inability to capture undiagnosed infections (Dasgupta et al., 2019), they nevertheless offer valuable insights for identifying priority populations and locations for intervention (Panneer et al., 2020). The integration of molecular network and spatial analyses therefore represents a powerful framework for designing more precise and effective HIV prevention strategies.

Several limitations should be acknowledged. First, although sequences were obtained from all 14 cities in Guangxi, sample sizes varied by location, which may introduce uncertainty in fine-scale phylogeographic inference for cities with smaller datasets. Therefore, inferred spatial dispersal patterns should be interpreted as statistically supported genetic connectivity rather than definitive transmission routes. Second, viral load measurements were collected during virologic failure after ART initiation and may not reflect infectiousness at the time of transmission; thus, viral load is interpreted as an indicator of persistent viral replication rather than transmission risk. Third, molecular transmission networks based on cross-sectional sequence data cannot reliably distinguish recent rapid transmission events from long-standing transmission chains, and network clustering should not be interpreted as direct evidence of recent transmission without complementary longitudinal data. Despite these limitations, the integrated molecular, phylogenetic, and spatial analyses provide valuable insights into HIV-1 transmission patterns and public health–relevant connectivity.

## Conclusion

This study presents the most comprehensive molecular epidemiological analysis of HIV-1 in Guangxi to date. By integrating molecular transmission networks, phylogenetic inference, and spatial analysis, we delineate subtype-specific connectivity patterns and identify key hub cities contributing to regional HIV-1 spread. Our findings underscore the need for precise, multidisciplinary HIV prevention strategies that combine individual-level risk reduction with geographically targeted interventions. These results provide robust scientific evidence to support HIV-1 control efforts with important implications for public health practice at both local and regional levels.

## Data Availability

The datasets presented in this study can be found in online repositories. The names of the repository/repositories and accession number(s) can be found here: NMDC, NMDC10020646, https://nmdc.cn/resource/genomics/project/detail/NMDC10020646.
