# Supplementary material for: Unraveling HIV-1 spread in Southwest China: a phylogenetic and molecular network approach
Source: Front Microbiol. 2026 Mar 9;17:1761072. doi: 10.3389/fmicb.2026.1761072 (PMC13007254; doi:10.3389/fmicb.2026.1761072)
Supplement: Supplementary file 1 [file Table_1.DOCX]

Supplementary materials


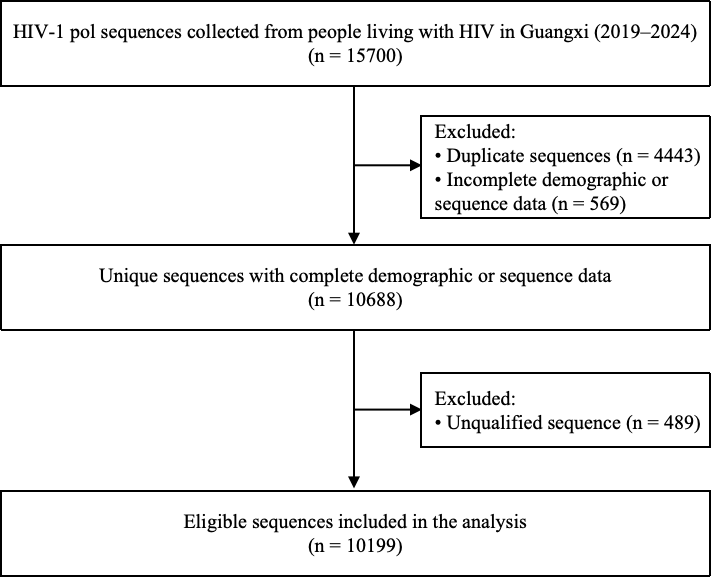


**Figure S1.** Sequences enrollment flowchart in the analysis.


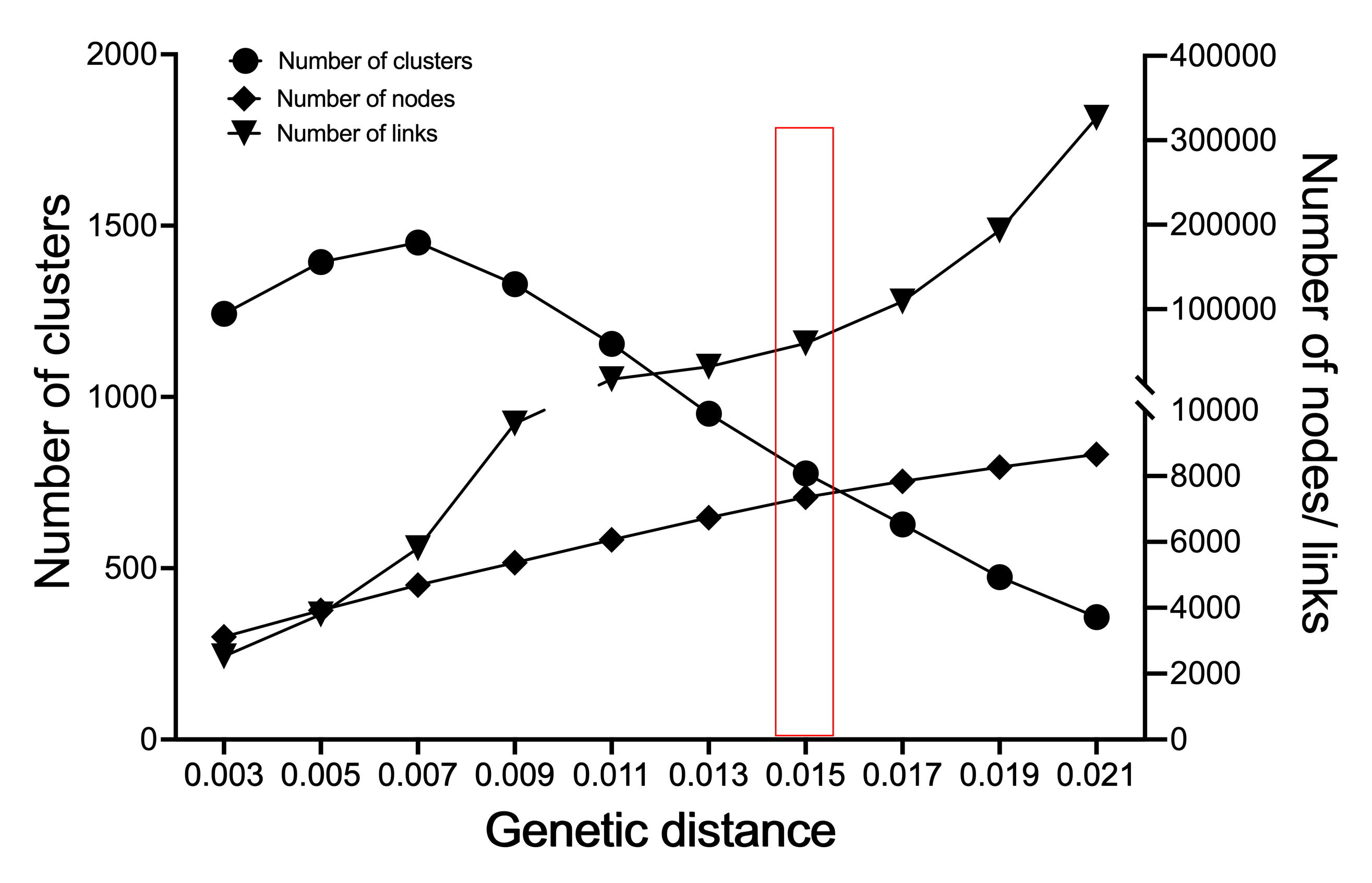


**Figure S2.** Selection of the optimal genetic distance (GD) threshold. Solid dotted curves represent the number of clusters at different GD thresholds. Solid rhomboid curves represent the number of nodes at different GD thresholds. Solid triangle curves represent the number of links at different GD thresholds. The red dashed line indicates the optimal GD threshold.


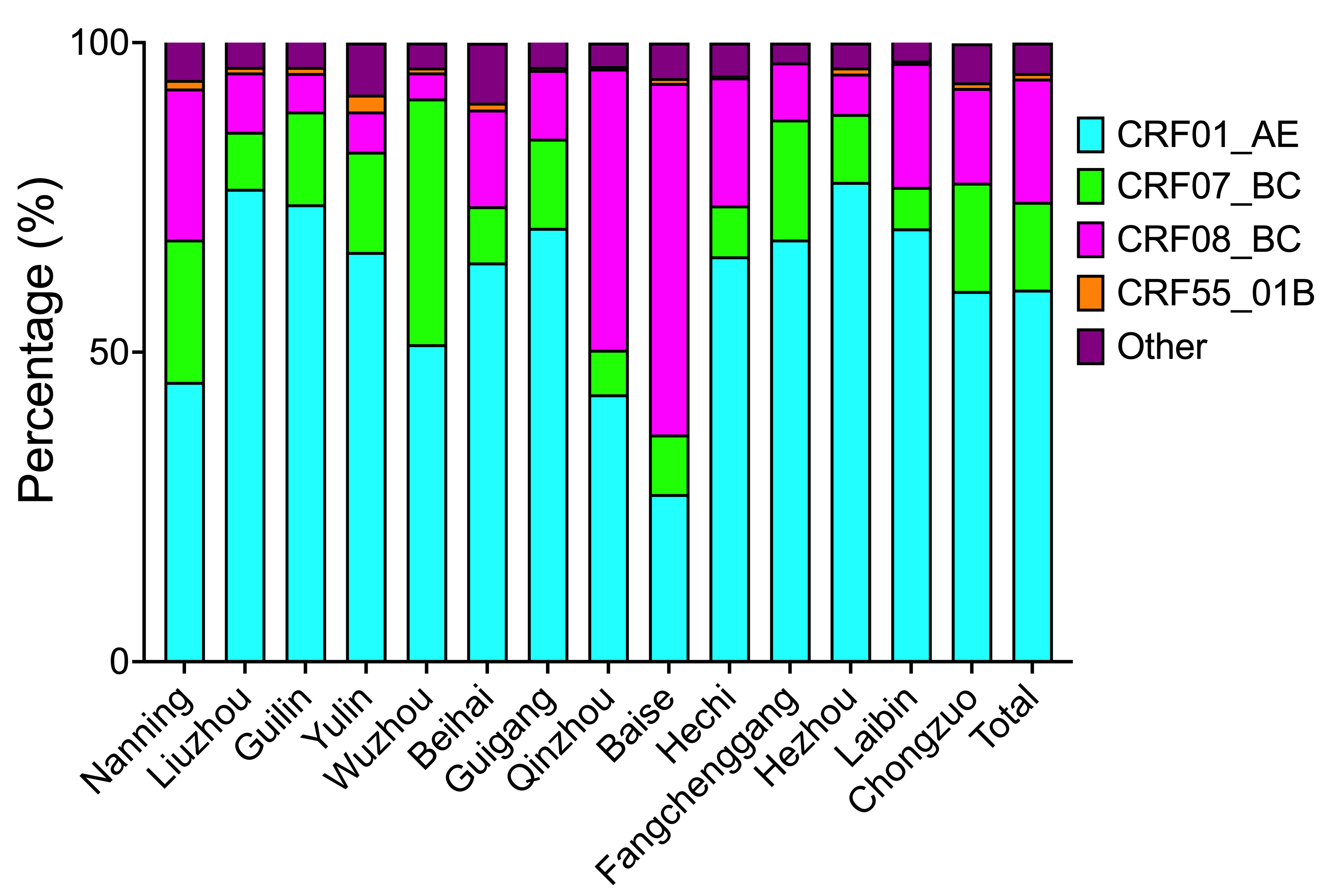


**Figure S3.** The distribution of subtypes among the 14 cities in Guangxi, China


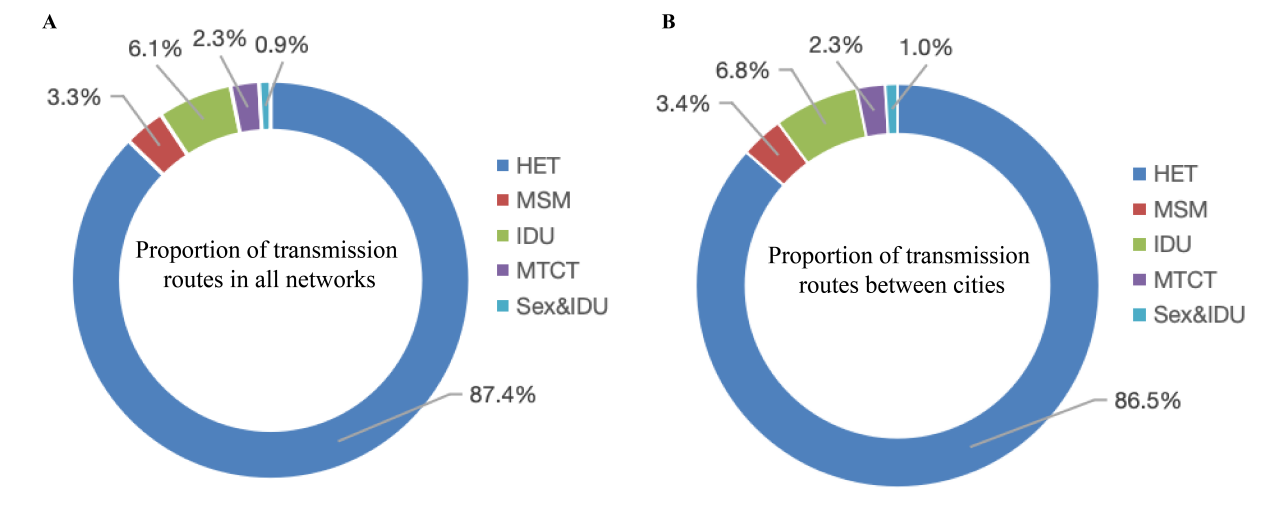


**Figure S4.** Proportions of the HIV-1 transmission routes among (A) all and (B) intercity links. A: Proportion of transmission routes in all networks; B: Proportion of transmission routes between cities. HET: Heterosexual transmission; MSM: Men who have sex with men; IDU: Intravenous drug use; Sex&IDU: Sexual contact and intravenous drug use; HTCT: Mother-to-child transmission.

**Table S1.** Clustering rate of different subtypes in each city of Guangxi, China

| City | Number | All | CRF01_AE | CRF07_BC | CRF08_BC | CRF55_01B | Other |
| --- | --- | --- | --- | --- | --- | --- | --- |
| Nanning | 1815 | 75.9% | 78.2% | 79.4% | 79.2% | 60.0% | 36.4% |
| Liuzhou | 1996 | 76.2% | 79.5% | 63.9% | 81.2% | 52.9% | 33.8% |
| Guilin | 827 | 76.1% | 80.5% | 66.1% | 70.6% | 75.0% | 39.4% |
| Yulin | 582 | 73.5% | 78.2% | 66.0% | 68.4% | 75.0% | 55.1% |
| Wuzhou | 378 | 81.5% | 87.1% | 78.7% | 81.3% | 66.7% | 40.0% |
| Beihai | 186 | 77.4% | 81.7% | 64.7% | 82.8% | 0.0% | 61.1% |
| Guigang | 605 | 76.2% | 81.6% | 74.7% | 71.6% | 0.0% | 8.3% |
| Qinzhou | 1099 | 72.6% | 66.1% | 86.1% | 81.6% | 25.0% | 19.0% |
| Baise | 627 | 70.5% | 70.6% | 63.3% | 73.9% | 60.0% | 50.0% |
| Hechi | 304 | 75.3% | 81.9% | 72.0% | 63.5% | 0.0% | 50.0% |
| Fangchenggang | 217 | 69.6% | 66.9% | 88.1% | 65.0% | 0.0% | 28.6% |
| Hezhou | 200 | 66.0% | 68.4% | 72.7% | 53.8% | 0.0% | 37.5% |
| Laibin | 827 | 84.3% | 86.9% | 74.5% | 87.9% | 33.3% | 28.0% |
| Chongzuo | 536 | 72.4% | 76.6% | 70.2% | 72.0% | 60.0% | 41.2% |
| Total | 10199 | 75.6% | 78.5% | 73.9% | 78.1% | 55.3% | 37.4% |

**Table S2.** Age composition of the PLWH in each city of Guangxi, China

| City | Number (%) | | |
| --- | --- | --- | --- |
|  | < 30 years (n=1683) | 30-49 years (n=3558) | ≥50 years (n=4958) |
| Nanning | 496 (27.3) | 506 (27.9) | 813 (44.8) |
| Liuzhou | 303 (15.2) | 727 (36.4) | 966 (48.4) |
| Guilin | 84 (10.2) | 242 (29.3) | 501 (60.6) |
| Yulin | 90 (15.5) | 171 (29.4) | 321 (55.2) |
| Wuzhou | 54 (14.3) | 122 (32.3) | 202 (53.4) |
| Beihai | 32 (17.2) | 72 (38.7) | 82 (44.1) |
| Guigang | 79 (13.1) | 197 (32.6) | 329 (54.4) |
| Qinzhou | 226 (20.6) | 460 (41.9) | 413 (37.6) |
| Baise | 58 (9.3) | 300 (47.8) | 269 (42.9) |
| Hechi | 32 (10.5) | 130 (42.8) | 142 (46.7) |
| Fangchenggang | 31 (14.3) | 76 (35.0) | 110 (50.7) |
| Hezhou | 49 (24.5) | 81 (40.5) | 70 (35.0) |
| Laibin | 84 (10.2) | 264 (31.9) | 479 (57.9) |
| Chongzuo | 65 (12.1) | 210 (39.2) | 261 (48.7) |
| Total | 1683 (16.5) | 3558 (34.9) | 4958 (48.6) |

**Table S3.** Sex composition of the PLWH in each city of Guangxi, China

| City | Number (%)† | |
| --- | --- | --- |
|  | Male | Female |
|  | (n=7312) | (n=2887) |
| Nanning | 1296 (71.4) | 519 (28.6) |
| Liuzhou | 1375 (68.9) | 621 (31.1) |
| Guilin | 597 (72.2) | 230 (27.8) |
| Yulin | 432 (74.2) | 150 (25.8) |
| Wuzhou | 291 (77.0) | 87 (23.0) |
| Beihai | 143 (76.9) | 43 (23.1) |
| Guigang | 429 (70.9) | 176 (29.1) |
| Qinzhou | 876 (79.7) | 223 (20.3) |
| Baise | 414 (66.0) | 213 (34.0) |
| Hechi | 207 (68.1) | 97 (31.9) |
| Fangchenggang | 141 (65.0) | 76 (35.0) |
| Hezhou | 148 (74.0) | 52 (26.0) |
| Laibin | 573 (69.3) | 254 (30.7) |
| Chongzuo | 390 (72.8) | 146 (27.2) |
| Total | 7312 (71.7) | 2887 (28.3) |

**Table S4.** Transmission route composition of the PLWH in each city of Guangxi, China

| City | Number (%) | | | | |
| --- | --- | --- | --- | --- | --- |
|  | HET | MSM | IDU | Sex&IDU | MTCT |
|  | (n=8767) | (n=449) | (n=642) | (n=103) | (n=238) |
| Nanning | 1372 (75.6) | 199 (11.0) | 83 (4.6) | 12 (0.7) | 149 (8.2) |
| Liuzhou | 1807 (90.5) | 56 (2.8) | 67 (3.4) | 6 (0.3) | 60 (3.0) |
| Guilin | 767 (92.7) | 45 (5.4) | 11 (1.3) | 3 (0.4) | 1 (0.1) |
| Yulin | 532 (91.4) | 22 (3.8) | 22 (3.8) | 4 (0.7) | 2 (0.3) |
| Wuzhou | 309 (81.7) | 12 (3.2) | 48 (12.7) | 4 (1.1) | 5 (1.3) |
| Beihai | 141 (75.8) | 9 (4.8) | 35 (18.8) | 1 (0.5) | 0 (0.0) |
| Guigang | 548 (90.6) | 19 (3.1) | 25 (4.1) | 10 (1.7) | 3 (0.5) |
| Qinzhou | 774 (70.4) | 15 (1.4) | 257 (23.4) | 47 (4.3) | 6 (0.5) |
| Baise | 591 (94.3) | 13 (2.1) | 20 (3.2) | 2 (0.3) | 1 (0.2) |
| Hechi | 291 (95.7) | 6 (2.0) | 6 (2.0) | 1 (0.3) | 0 (0.0) |
| Fangchenggang | 209 (96.3) | 4 (1.8) | 2 (0.9) | 2 (0.9) | 0 (0.0) |
| Hezhou | 152 (76.0) | 9 (4.5) | 26 (13.0) | 2 (1.0) | 11 (5.5) |
| Laibin | 793 (95.9) | 18 (2.2) | 13 (1.6) | 3 (0.4) | 0 (0.0) |
| Chongzuo | 481 (89.7) | 22 (4.1) | 27 (5.0) | 6 (1.1) | 0 (0.0) |
| Total | 8767 (86.0) | 449 (4.4) | 642 (6.3) | 103 (1.0) | 238 (2.3) |

**Table S5.** Number of intercity links and intracity links between PLWH in each city of Guangxi, China

| City | All | | CRF01_AE | | CRF07_BC | | CRF08_BC | | CRF55_01B | | Other | |
| --- | --- | --- | --- | --- | --- | --- | --- | --- | --- | --- | --- | --- |
|  | Total links | Intercity Frequency (%) | Total links | Intercity Frequency (%) | Total links | Intercity Frequency (%) | Total links | Intercity Frequency (%) | Total links | Intercity Frequency (%) | Total links | Intercity Frequency (%) |
| Nanning | 1941 | 48.9 | 882 | 52.0 | 492 | 43.9 | 498 | 48.4 | 19 | 47.4 | 50 | 50.0 |
| Liuzhou | 2227 | 46.1 | 1815 | 44.2 | 146 | 57.5 | 222 | 53.2 | 11 | 63.6 | 33 | 45.5 |
| Guilin | 914 | 45.7 | 741 | 45.5 | 116 | 43.1 | 39 | 43.6 | 4 | 100.0 | 14 | 71.4 |
| Yulin | 593 | 43.3 | 442 | 43.7 | 70 | 44.3 | 26 | 42.3 | 13 | 46.2 | 42 | 38.1 |
| Wuzhou | 411 | 51.1 | 219 | 50.7 | 145 | 57.2 | 18 | 66.7 | 0 | - | 8 | 50.0 |
| Beihai | 175 | 41.1 | 120 | 37.5 | 15 | 60.0 | 28 | 57.1 | 0 | - | 12 | 16.7 |
| Guigang | 667 | 49.8 | 503 | 48.9 | 98 | 52.0 | 64 | 51.6 | 0 | - | 2 | 100.0 |
| Qinzhou | 1023 | 47.8 | 346 | 55.2 | 84 | 56.0 | 580 | 42.1 | 1 | 100.0 | 12 | 50.0 |
| Baise | 584 | 44.9 | 155 | 45.8 | 38 | 50.0 | 365 | 43.6 | 1 | 100.0 | 25 | 48.0 |
| Hechi | 325 | 52.6 | 243 | 49.4 | 19 | 68.4 | 55 | 58.2 | 0 | - | 8 | 75.0 |
| Fangchenggang | 183 | 52.5 | 114 | 53.5 | 55 | 41.8 | 12 | 83.3 | 0 | - | 2 | 100.0 |
| Hezhou | 159 | 55.3 | 130 | 53.1 | 19 | 78.9 | 7 | 42.9 | 0 | - | 3 | 33.3 |
| Laibin | 1053 | 52.9 | 768 | 52.9 | 47 | 51.1 | 230 | 52.6 | 1 | 100.0 | 7 | 71.4 |
| Chongzuo | 503 | 51.7 | 304 | 50.0 | 108 | 52.8 | 71 | 54.9 | 3 | 100.0 | 17 | 52.9 |
| Total | 10758 | 48.2 | 6782 | 48.1 | 1452 | 49.7 | 2215 | 47.7 | 53 | 60.4 | 235 | 48.9 |

**Table S6.** Overview of well-supported HIV migration events across cities of Guangxi, China

| HIV-1 Subtypes | From | To | Bayes factor | Posterior probability |
| --- | --- | --- | --- | --- |
| CRF01_AE | baise | liuzhou | 113 | 0.90 |
|  | chongzuo | fangchenggang | 456 | 0.97 |
|  | guigang | wuzhou | 22115 | 1.00 |
|  | guilin | nanning | 795 | 0.98 |
|  | hezhou | wuzhou | 110622 | 1.00 |
|  | qinzhou | wuzhou | 110622 | 1.00 |
|  | qinzhou | yulin | 110622 | 1.00 |
|  | qinzhou | beihai | 110622 | 1.00 |
|  | qinzhou | baise | 576 | 0.98 |
|  | qinzhou | baise | 110622 | 1.00 |
|  | qinzhou | chongzuo | 110622 | 1.00 |
|  | qinzhou | fangchenggang | 110622 | 1.00 |
|  | qinzhou | guigang | 110622 | 1.00 |
|  | yulin | guigang | 110622 | 1.00 |
|  | hezhou | guilin | 166 | 0.93 |
|  | qinzhou | guilin | 110622 | 1.00 |
|  | liuzhou | hechi | 110622 | 1.00 |
|  | qinzhou | hechi | 110622 | 1.00 |
|  | qinzhou | hezhou | 110622 | 1.00 |
|  | liuzhou | laibin | 110622 | 1.00 |
|  | qinzhou | laibin | 110622 | 1.00 |
|  | qinzhou | liuzhou | 110622 | 1.00 |
|  | qinzhou | nanning | 110622 | 1.00 |
|  | yulin | qinzhou | 7890 | 1.00 |
| CRF07_BC |  |  |  |  |
|  | chongzuo | liuzhou | 139 | 0.92 |
|  | fangchenguigangang | qinzhou | 358 | 0.97 |
|  | guigang | laibin | 1406 | 0.99 |
|  | guigang | liuzhou | 1245 | 0.99 |
|  | guilin | hezhou | 1105 | 0.99 |
|  | nanning | qinzhou | 110622 | 1.00 |
|  | nanning | wuzhou | 110622 | 1.00 |
|  | nanning | yulin | 110622 | 1.00 |
|  | wuzhou | yulin | 110622 | 1.00 |
|  | laibin | beihai | 745 | 0.98 |
|  | nanning | beihai | 18427 | 1.00 |
|  | chongzuo | baise | 120 | 0.91 |
|  | nanning | baise | 110622 | 1.00 |
|  | nanning | chongzuo | 110622 | 1.00 |
|  | nanning | fangchenguigangang | 55305 | 1.00 |
|  | nanning | guigang | 110622 | 1.00 |
|  | wuzhou | guigang | 880 | 0.99 |
|  | yulin | guigang | 115 | 0.90 |
|  | liuzhou | guilin | 110622 | 1.00 |
|  | nanning | guilin | 110622 | 1.00 |
|  | nanning | hechi | 110622 | 1.00 |
|  | nanning | hezhou | 110622 | 1.00 |
|  | wuzhou | hezhou | 110622 | 1.00 |
|  | liuzhou | laibin | 1744 | 0.99 |
|  | nanning | laibin | 110622 | 1.00 |
|  | nanning | liuzhou | 110622 | 1.00 |
|  | wuzhou | liuzhou | 859 | 0.99 |
|  | wuzhou | nanning | 110622 | 1.00 |
|  | wuzhou | qinzhou | 193 | 0.94 |
| CRF08_BC |  |  |  |  |
|  | baise | fangchenguigangang | 159 | 0.93 |
|  | baise | hechi | 16677 | 1.00 |
|  | baise | nanning | 83433 | 1.00 |
|  | chongzuo | nanning | 153 | 0.93 |
|  | hechi | liuzhou | 130 | 0.91 |
|  | laibin | liuzhou | 83433 | 1.00 |
|  | nanning | qinzhou | 83433 | 1.00 |
|  | nanning | wuzhou | 83433 | 1.00 |
|  | nanning | yulin | 83433 | 1.00 |
|  | qinzhou | beihai | 83433 | 1.00 |
|  | nanning | baise | 83433 | 1.00 |
|  | qinzhou | baise | 83433 | 1.00 |
|  | nanning | chongzuo | 83433 | 1.00 |
|  | qinzhou | chongzuo | 1426 | 0.99 |
|  | qinzhou | fangchenguigangang | 4380 | 1.00 |
|  | nanning | guigang | 83433 | 1.00 |
|  | nanning | guilin | 83433 | 1.00 |
|  | nanning | hechi | 83433 | 1.00 |
|  | nanning | hezhou | 83433 | 1.00 |
|  | liuzhou | laibin | 83433 | 1.00 |
|  | nanning | laibin | 83433 | 1.00 |
|  | nanning | liuzhou | 83433 | 1.00 |
|  | qinzhou | liuzhou | 83433 | 1.00 |
|  | qinzhou | nanning | 83433 | 1.00 |
| CRF55_01B |  |  |  |  |
|  | nanning | yulin | 203222 | 1.00 |
|  | nanning | chongzuo | 123 | 0.92 |
|  | nanning | guilin | 2128 | 0.99 |
|  | yulin | laibin | 188 | 0.94 |
|  | nanning | liuzhou | 203222 | 1.00 |

Only results with a Bayes factor (BF) ≥3 and posterior probability support ≥0.9 are presented.
